# The “Instinct” of Imagination. A Neuro-Ethological Approach to the Evolution of the Reflective Mind and Its Application to Psychotherapy

**DOI:** 10.3389/fnhum.2018.00522

**Published:** 2019-01-23

**Authors:** Antonio Alcaro, Stefano Carta

**Affiliations:** ^1^Department of Psychology, Sapienza University of Rome, Rome, Italy; ^2^Department of Pedagogy, Psychology, and Philosophy, Università degli Studi di Cagliari, Cagliari, Italy

**Keywords:** default mode network, self, affective, dream (images), mentalization, emotion, consciousness, noetic

## Abstract

Recent neuro-psychoanalytic literature has emphasized the view that our subjective identity rests on ancient subcortical neuro-psychic processes expressing unthinking forms of experience, which are “affectively intense without being known” (Solms and Panksepp, 2012). Devoid of internal representations, the emotional states of our “core-Self” (Panksepp, 1998b) are entirely “projected” towards the external world and tend to be discharged through instinctual action-patterns. However, due to the close connections between the subcortical and the cortical midline brain, the emotional drives may also find a way to be reflected within an intrinsic self-referential processing, evident when the organism is not actively engaged with the external world. Thanks to such endogenous functioning, the core-Self emotional dispositions are not overtly executed, but they are organized within coherent dynamic mental structures, called “feeling-toned complexes” by C. G. Jung and “unconscious phantasies” by Melanie Klein. The intrinsic self-referential dynamism of the “brainmind” originated from REM sleep arousal and then evolved in the resting-state activity of a complex of cortico-limbic midline brain structures (CMS), also called Default Mode Network (DMN). From our neuro-ethological perspective, it is sustained by an “introverted” SEEKING activity leading to the subjective exploration of internally constructed virtual scenarios. This “mind wandering” function, implicated in dreaming, fantasy processing, remembering and thinking, is the essence of the imaginative function and constitutes the first form of reflection, where intentions and drives gain a primordial form of conscious (but not self-conscious) representation. During postnatal development, this original (“archetypal”) imaginative function is slowly attuned in a relational “transitional” space and may be expressed first in non-verbal and eventually in abstract-verbal social communicative patterns. Our view has noticeable implications for psychotherapy. Instead of trying to directly modify interpersonal, extrinsic relationships (a top-down approach), dysfunctional emotional-relational patterns may be modified by a process in which the patient is helped to let-go of the perceived feeling-objects in favor of an immersion, *via* the actual feeling, from the superficial level of perception towards a void feeling-state, empty of images. Only starting from this “anoetic” feeling-state, the deep imaginal creative and re-structuring self-referential activity may be reactivated by a process of spontaneous imagination.

## Introduction

Most scholars today consider subjectivity (personal experience) the result of an individual historical process and, therefore, as an acquisition of human development. In particular, the emergence of a mental life and of a sense of “self” have been associated with emotional and cognitive skills that permit to the child to reflect upon his/her experience (Damasio, 2010). Such essential function, called “mentalization” or “reflective function,” is usually considered a product of the attachment relationship (Stern, 1985; Schore, 1994; Fonagy and Target, 1997; Fonagy et al., 2002). Cognitive psychologists, psychoanalysts and neuroscientists are in agreement that the child *learns* to have a subjective mental life and to recognize it as his own by internalizing experiences and aspects of the attachment relationship, which are dependent on the self-reflective capacity of the caregiver (Bowlby, 1969/1982; Bretherton and Munholland, 1999).

While recognizing the importance of such theories, we think that strict developmental views fail to consider that newborns are provided by an innate potential endogenous mental activity where the intersubjective environment is subjectively experienced and represented. Indeed, neurodevelopmental studies have shown that human embryos already possess an intrinsic brain dynamism typical of REM sleep, that appears before non-REM sleep and active waking and that is largely preponderant in the last trimester of pregnancy (Birnholz, 1981). Interestingly, REM sleep leads to the endogenous activation of a complex of subcortical midline structures (SCMS) and cortical midline brain structures (CMS) involved in highly emotional self-referential processing. Moreover, recent neurometabolic studies showed that CMS show high levels of metabolic activity not only during REM-sleep, but also in resting wakefulness, when the organism is not engaged with the environment (Raichle, 2015).

The endogenous self-referential processing of the CMS has been related to an intrinsic virtual-models generator function, through which the organism forms an inferential knowledge about the structure of (its) reality (Hobson and Friston, 2012). During REM sleep, this inferential process may be subjectively experienced in the form of perceptual-like images that we call dreams, and that express a primordial form of consciousness, or proto-consciousness (Hobson, 2009). During resting wakefulness, it may be subjectively experienced as an internal flux of imagines and thoughts (Carhart-Harris and Friston, 2010; Schacter et al., 2012; Agnati et al., 2013; Fox et al., 2013) that clearly resembles what William James described as the “stream of consciousness”:

“*As we take […] a general view of the wonderful stream of our consciousness, what strikes us first is this different pace of its parts. Like a bird’s life, it seems to be made of an alternation of flights and perchings […] The resting-places […] can be held before the mind for an indefinite time […] The places of flight … obtain between the matters contemplated in the periods of comparative rest” (James, 1890)*.

Therefore, following such neuroscientific paths and in accordance with Jung’s Analytical Psychology (Jung, 1937), we believe that the so-called “reflective function” is grafted in an embryonic form of mental activity, that lays on the “off-line,” resting-state activity of the brain. Flowing from this instinct for reflection, the intrinsic emotional dispositions of the living organism are maintained in a “potential state” and rather than expressing itself in actions (motor or physiological) they fit back upon themselves and eventually establish a subjective psychic dimension (Jung, 1937; Shamdasani, 2003). Such essential self-referential function, evident in dreaming, remembering, thinking and fantasy processing, generates images as the first phenomenological building blocks of our nature as wholly psychic animals. Indeed, as suggested by Jung, “everything of which we are conscious is an image, and that image is psyche … [which] is a world in which the ego is contained” (Jung, 1967, p.75).

Therefore, within this contribute we present a neuro-ethological viewpoint (MacLean, 1990; Panksepp, 1998b; Panksepp and Biven, 2012) that integrates human and animal evidences showing how the “imaginative function” have evolved in terrestrial vertebrates and which kind of brain processes are related to it. In the final part of the manuscript, we discuss some implication of our perspective for clinical psychology and psychotherapy.

## The DMN Resting-State Activity and Self-Referential Processing

In 1997, Shulman et al. (1997) published a meta-analytic study showing brain areas with increased blood flow during passive viewing of a stimulus array relative to that during active tasks. The brain areas showing high metabolism at rest belong to a complex of CMS that are tightly connected form an anatomic and functional point of view and have been defined as the Default Mode Network (DMN; Raichle et al., 2001; Raichle and Snyder, 2007). As shown by Rahicle “the brain’s DMN consists of discrete, bilateral and symmetrical cortical areas, in the medial and lateral parietal, medial prefrontal, and medial and lateral temporal cortices of the human, nonhuman primate, cat, and rodent brains” (Raichle, 2015, p. 433).

It has been showed that the DMN is characterized by high intrinsic activity during resting states with visual fixating or with eye closed that decreases when subjects engage in goal-directed tasks (Gusnard et al., 2001; Raichle et al., 2001; Greicius et al., 2003). As a consequence, the DMN has been linked to internally-oriented mental processes (Mason et al., 2007) and its functioning is considered in contrast to the dorsal attention network (DAN), that is recruited when attention is directed towards the external world^1^ (Corbetta et al., 2008). Moreover, the DMN resting-state activity is particularly evident during REM sleep, an evolutionary old state of the brain characterized by intense neurophysiological arousal accompanied by the organism’s isolation from the external world (Nir and Tononi, 2010).

Since the great majority of brain metabolism is directed to aliment the DMN resting-state activity, such disproportionate energy consumption not employed in active engagement with the environment has led to an analogy with the dark energy of cosmology. As Zhang and Raichle (Zhang and Raichle, 2010, p. 15) write:

“*Like our cosmos, the brain also has its own ‘dark energy.’ Indeed, ‘visible’ elements of brain activity—neuronal responses to environmentally driven demands—account for less than 5% of the brain’s energy budget, leaving the majority devoted to intrinsic neuronal signaling” (Zhang and Raichle, 2010, p. 15)*.

Therefore, an open question among researchers is to ascertain which psychological functions are related to such intrinsic functioning of the brain. In the attempt to answer to this question, an increasing body of human studies has shown that the DMN are crucial for self-specific processing, that is the elaboration of stimuli and activities related to the self^2^ (Northoff, 2011, 2013, 2016). For example, functional brain imaging studies show that DMN is strongly engaged when personal relevant stimuli, such as person’s name, are presented compared with non-self-related ones, like another person’s name (Qin and Northoff, 2011). Most surprisingly, DMN activity does not change if we compare self-related activity and resting-state activity (D’Argembeau et al., 2005). This “rest–self overlap” (Northoff, 2016) strongly suggests that resting-state activity is in some way related to the processing of self-relevant information.

Studies of the self-referential DMN activity have radically changed our understanding of the brain. Until the 1990’s, research in cognitive psychology and neuroscience was dominated by a task-centric view of mental functioning and emphasized the view of the “brainmind” as an organ reacting to enteroceptive and/or exteroceptive stimuli. However, the massive presence of endogenous activity implies a tendency to form self-organized dynamic patterns (Northoff, 2013, 2014a,b, 2015a,b, 2016, 2018; Northoff and Stanghellini, 2016; but see also Brown, 2002; Llinás, 2002). This also implies that the “brainmind” does not merely react to stimuli, but that it integrates them within its endogenous “intrinsic” functioning (Northoff et al., 2010a; Northoff and Stanghellini, 2016; Northoff, 2018).

Under a Jungian light, the endogenous mental activity (the Self) is formed by potential psychic organizers that give to outer objects their emotional and cognitive value/meaning. Such organizers derive from archetypal schemas that possess a general validity for interpreting the perceived stimuli, which will progressively match with outer experiences and perceptions in order to form personalized complexes (see next paragraph). After Jung, also Melanie Klein’s unconscious phantasies (Klein, 1921, 1952/2018; Isaacs, 1948), or Winnicott (1967) “conceived objects,” expressed the idea of endogenous psychological structures that match with perceived objects, and that are eventually stored under the form of memories belonging to coherent episodes and narratives^3^.

## The Affective-Emotional Ground of the DMN Self-Referential Activity

Recent neuroscientific evidences show that the degree of self-relatedness depends on the functioning of a core ventral portion of the DMN (D’Argembeau et al., 2005), which is specifically involved in carrying the emotional-affective ground within internally-oriented mental activity (Christoff et al., 2016). In other words, the ventral portion of the DMN “constrains” the imaginative activity within specific orbits of meaning centered on characteristic emotional feelings or moods.

The ventral-core part of the CMS includes the ventromedial prefrontal cortex (VMPFC), the medial orbitofrontal cortex (MOFC), and the sub- and pregenual part of the anterior cingulate cortex (PACC). These regions are densely connected with subcortical regions (brain stem, midbrain, and basal forebrain), as well as with the amygdala, the basal ganglia (striatum and nucleus accumbens), and all primary exteroceptive sensory modalities (Northoff et al., 2006; Christoff et al., 2016). In particular, the affective-emotional grounding exerted by the ventral portion of the DMN depends on the strict connections with a complex of SCMS (Northoff et al., 2006) involved in basic visceral-homeostatic regulation as well as in the emergence of instinctual drives and basic emotional dispositions (Panksepp, 1998a,b, 2005, 2010, 2011; Damasio, 1999; Denton, 2006; Alcaro et al., 2017).

In consideration of their essential functions, the SCMS have been called the “core-Self” (Panksepp, 1998b), or “proto-Self” (Damasio, 1999). They have been implicated in the emergence of a primary form of affective consciousness, characterized by moods, somato-visceral states and basic emotional feelings, which constitute the first form of self-orientation in the world (Panksepp, 1998b; Damasio, 1999; Merker, 2007; Solms and Panksepp, 2012; Alcaro and Panksepp, 2014; Alcaro et al., 2017). Such primordial form of “anoetic” awareness has been defined as “the rudimentary state of autonomic awareness […], with a fundamental form of first-person “self-experience” which relies on affective experiential states and raw sensory and perceptual mental existences” (Vandekerckhove and Panksepp, 2009, p. 1).

By anchoring its activity within specific affective routs, the ventral portion of the DMN unfolds an essential settling function for the emergence of a stable sense of self and of external reality. Interestingly, clinical observations demonstrated that patients with lesions in ventral portion of the DMN present a deeply disorganized, psychotic-like mental activity (Solms and Solms, 2000) and remain unable to develop a coherent model of their own self (Damasio, 1999; Schore, 2003, 2009). In our opinion, this may be viewed as the consequence of the disconnection between the DMN and the SCMS. In such case, the imaginative function loses its core-Self affective ground, essential for its coherence, integration, stability, and self-relatedness.

On the other hand, when the power of affect is excessively high, the focus of mental activity may be strongly restricted. This gives rise to rumination, where fixed thoughts or imagines are compulsively produced and dominate the mental field. This phenomenon is particularly evident in depression, a pathological mental state characterized by hyperemotional ruminative thoughts and by increased resting-state activity in the cortical-subcortical midline brain structures (Alcaro et al., 2010; Northoff et al., 2011).

Surprisingly, the absolute relevance of affective processes for self-relatedness was grasped long time ago by C. G. Jung, who wrote that:

“*Every psychic process has a value quality attached to it, namely its feeling-tone. This indicates the degree to which the subject is affected by the process or how much it means to him (in so far as the process reaches consciousness at all). It is through the ‘affect’ that the subject becomes involved and so comes to feel the whole weight of reality” (Jung, 1959: para. 61)*.

According to Jung, affects not only carry an intrinsic subjective quality, but also an essential integrative property. Indeed, he thought that mental life is organized around specific dynamic structures, called “feeling-toned complexes,” that gather together different mental contents on the base of a common affective state (see also Wilkinson, 2006). Each complex is unified by the same affect, which defines its core of meaning, and organizes experience, perception, phantasy and thought around a central theme. For example, a particular complex of inferiority may be made by a constellation of memories, thoughts and phantasies related to the lack of self-worth, a doubt and uncertainty about oneself, and feelings of not measuring up to standards.

The Jungian theory of the feeling-toned complexes is an elaboration of the work of Pierre Janet on the autonomous fixed ideas. According to Janet (1889), fixed ideas are mental images or thoughts that have a high emotional charge and take on exaggerated proportions, so they may not be normally integrated within the ego-consciousness and become isolated from the habitual personality, creating dissociated states of the mind (Monahan, 2009). However, in contrast to the original theory of Janet, Jung sustained that the dissociative aspect of complexes is usually reversible, so they may be much or more integrated according to the momentary situation. Only in severe mental pathologies, such as psychoses, certain complexes are permanently dissociated from the conscious ego and the personality become fragmented. Such disconnection exposes the subject to the powerful and not-modulated activity of “his” complexes, and the subject is anancastically forced to react to his endogenous affective, archaic images, almost devoid of voluntary agency and proper working memory.

## The Essence of the Imaginative Function: Mind Wandering as Introverted Seeking

The highly affective self-referential resting-state DMN activity may act in a total unconscious way, influencing the individual attitude towards its environment and the subjective (affective) meaning given to external experiences. Such unconscious processing has been described by clinical psychologists by the concept of “implicit unrepressed unconscious” (Fonagy et al., 2002), or of “internal working models” (Bowlby, 1969/1982; Stern, 1985). However, the self-referential processing of the brain may also become accessible to subjective awareness by a process of active imagination that enlightens the intrinsic dispositions of the Self and its prediction models (Alcaro and Panksepp, 2014). The emerging conscious representations refer to objects that have not actually been perceived by the exteroceptive senses, but that are subjectively built in order to express and represent the intrinsic dispositions of the living organism (instincts, drives, habits, emotions) and their connection with ongoing predictive operative working models (see Appendix 1 for a definition of images and image schema).

When self-referential processing accedes to subjective awareness, our mental field is populated by perceptual-like exteroceptive images with predominant (but not exclusive) visuo-spatial features. The product of this reflective instinct is a pouring out of images (and thoughts) that, like jets of clear water from the rocks of a mountain’s stream, are born in a borderland between the unconscious and the conscious, between the body and the mind, and whose substratum is that of raw emotions.

In accordance with such idea, contemporary neuroscientists start to look at DMN resting-state activity in relation to the spontaneous and free-floating flux of conscious images and thoughts implicated in remembering past events, anticipating future events or simply in simulating fantastic or virtual scenarios (Carhart-Harris and Friston, 2010; Schacter et al., 2012; Agnati et al., 2013; Fox et al., 2013; Christoff et al., 2016). Some of them refers to such spontaneous imaginative function with the term “mind wandering” (Mason et al., 2007; Fox et al., 2013; Christoff et al., 2016), defined as the tendency of moving “higher and tighter without fixed course or certain aim” (Simpson, 1989). More specifically, mind wandering is a mental exploration characterized by spontaneity, internal orientation, high propensity to explore, positive affects, freedom from constraints, creativity and high entropy^4^ (Carhart-Harris et al., 2014; Christoff et al., 2016).

Mind wandering has been specifically related to the activity of the medio-temporal-lobe (MTL), a set of brain areas centered on the hippocampus and the parahippocampal complex that constitute the oldest component of the DMN and whose antecedents have been found in the medial cortex of reptiles (Reiter et al., 2017). The MTL is implicated in navigating through the world (both physically and mentally) and in the formation of complex and abstract representations necessary for spatio-temporal orientation, declarative memory, contextual learning, creative thinking and social behaviors (Reiter et al., 2017).

It has then been proposed that the MTL deserves a “self-projective” function, through which the individual travels across a virtual dimension, imaging what has happened, what may happen or what could never happen. Due to such self-projective function, the MTL is implicated in forming new contextual associations, remembering past episodic events, or imaging virtual, novel and potential future scenarios (Buckner and Carroll, 2007; Buckner et al., 2008; Buckner, 2010; Maguire and Hassabis, 2011).

In our neuro-ethological perspective, the self-projective exploratory function mediated by the MTL is supported by the expression of a basic emotional drive, called the SEEKING disposition, whose neural substrates are centered on the ascending mesolimbic dopaminergic system and its forebrain projection areas (Alcaro et al., 2007; Alcaro and Panksepp, 2011, 2014). The SEEKING system is an intrinsic psycho-behavioral function of the brain that evolved to induce organisms to explore and to search for all varieties of life-supporting stimuli (Ikemoto and Panksepp, 1999; Alcaro et al., 2007; Alcaro and Panksepp, 2011). Its activation “changes the individual’s attitude towards the environment, promoting an energized appetitive disposition, which unconditionally promotes exploration and foraging for resources, and creates expectancy states that allow animals to anticipate the presence of future rewards” (Alcaro and Panksepp, 2011, p. 1805).

Interestingly, animal studies have shown that the exploratory SEEKING activity is accompanied by the emergence of certain oscillatory rhythms in some forebrain areas belonging to the DMN (for a review, see Alcaro and Panksepp, 2011). More specifically, orienting and exploratory movements are associated with theta oscillations in the hippocampal complex, coupled with gamma oscillations within the ventro-medial frontal cortex and basal ganglia circuits (for a review, see Alcaro and Panksepp, 2011). These neurodynamic patterns seem related to the tendency to move, explore and approach specific sources of stimulation and to the internal attitude to be open and trusty towards novel and unpredictable experiences (Alcaro and Panksepp, 2011).

Moreover, it is also presumable that the same SEEKING neurodynamic patterns are activated also when the exploratory movements are directed towards imaginal objects or thoughts. In accordance with such a view, studies of patients with brain lesions have confirmed that the ventro-medial quadrant of the frontal lobe, that is rich in projections from the SEEKING system, is necessary for dream generation and mind wandering (Solms, 1997, 2000). Moreover, the dopaminergic-SEEKING system is thought to contribute to creativity, in particular divergent thinking, cognitive flexibility, innovative insights, and associative thinking (Flaherty, 2005; Chermahini and Hommel, 2010; Takeuchi et al., 2010; Zabelina et al., 2016; Boot et al., 2017).

As suggested in a previous article, the exploratory SEEKING drive may also have an introverted direction that “permits the channeling of SEEKING not only towards external objects of perception, but also towards internalized mental representations of subjective environments” (Alcaro and Panksepp, 2011, p. 1809). This phenomenon is evident when the categories of stimuli and configurations represented within the MTL do not refer to an external space, but instead to the internally oriented psychological world.

As already indicated in the previous paragraph, the self-referential DMN resting-state activity is usually constrained within specific orbits or “basins of attraction” (Carhart-Harris et al., 2014; Christoff et al., 2016). In particular, some researcher refers to “automatic constraints” to indicate the involuntary influence exerted by feeling-toned complexes elaborated within the ventral portion of the DMN and the SCMS^5^ (Christoff et al., 2016). The expression of the automatic emotional constraints may be registered and represented within the MTL, which condenses the operative procedural functioning of the DMN into abstract configurations (image schema). When such configurations become the objects of mind wandering, the unconscious self-referential process elaborated within the DMN is enlightened and transformed into a noetic conscious flux of mental representations (Alcaro and Panksepp, 2014). As shown in the next paragraph, such function is maximally evident during REM sleep dreaming.

## Dreaming as the Archetypal Form of Mind Wandering

It has been widely underlined that REM sleep’s dreaming constitute the purest and most archaic phenomenal expression of all the possible forms of mind-wandering (Christoff et al., 2016). This claim is perfectly in line with the psychoanalytic assumption that dreams are the “royal road to the unconscious,” since they reveal clearly the functioning of our ancestral and primary-process mental activity (Freud, 1900; Jung, 1967).

Interestingly, recent experimental evidences demonstrated that also reptiles show neurophysiological and behavioral correlates of the REM phase during sleep (Shein-Idelson et al., 2010). This evidence suggests the possibility that that dreaming may be not an exclusive characteristic of humans, but instead an instinctual function of the vertebrate brain. Interestingly, during the REM phase, endothermic animals lose their thermoregulatory capacity, so the body is left without its usual metabolic controls, while the brain instead becomes metabolically hyperactive especially in certain MTL regions (Cerri et al., 2017). The “regression” to a metabolic pattern similar to the ectothermic state supports the view than dreams represent an evolutionary archaic mode of functioning of the brainmind (Panksepp, 1998b) and express a primary form of consciousness, or protoconsciousness (Hobson, 2009). Such view also fit with the fact that the brainstem nuclei controlling the REM phase are evolutionary oldest that those controlling slow wave sleep and active wakefulness (Panksepp, 1998b). Moreover, neurodevelopmental studies show that REM sleep appears before non-REM sleep and active waking in the human embryos, and that REM sleep is largely preponderant in the last trimester of pregnancy, decreasing progressively after birth (Birnholz, 1981).

The idea that REM-sleep dreaming represents an archaic and unconstrained form of mind wandering is supported by the evidences showing that the REM-phase is characterized by:

1. a robust (“bursting”) activation of the SEEKING-mesolimbic dopamine system (Hartman et al., 1980; Gottesman, 1999; Dahan et al., 2007; Perogamvros and Schwartz, 2012, 2015), which may have something in common with that observed in florid schizophrenia (dominated by positive symptoms such as hallucinations, delusions, hyper-vigilant states, etc.; Solms, 1997);
2. a maximum neurometabolic activity in the MTL, the oldest part of the DMN that endotherms share with reptiles (Christoff et al., 2016);
3. a major independence of MTL activity from the influence exerted by other DMN areas (Carhart-Harris et al., 2014);
4. a deactivation of cortical areas that exert deliberate control over mental functioning and that are involved in self-monitoring, such as the orbitofrontal cortex, the posterior cingulate cortex, the precuneus, the dorsolateral prefrontal cortex, and the inferior parietal cortex (Nir and Tononi, 2010; Christoff et al., 2016);
5. a profound inhibition of the primary perceptual and motor areas.

In relation to the neurophysiological peculiarities of REM-sleep, REM-type dreaming expresses several phenomenological differences with waking consciousness (for a review, see Nir and Tononi, 2010). First of all, dreams are often characterized by increased emotional arousal and affective involvement (Schredl, 2010). Such increased emotionality in REM sleep is related to hyper-activation of the highly emotional limbic brain (for a review, see Nir and Tononi, 2010) and, in particular, of the SCMS, where all the main basic emotional systems are located (Panksepp, 1998a,b; Alcaro et al., 2017). Interestingly, also animals dream highly emotional experiences, since cats with lesions in neural areas responsible for muscle atony during REM, show complex emotional behaviors of attack, defense and exploration (Sastre and Jouvet, 1979). Similarly, highly emotional behaviors are also frequent in subjects with REM sleep behavior disorder, who act out their dreams (Oudiette et al., 2009). Moreover, experimental evidences in humans demonstrated that after the 13th week of gestation and during the neonatal period smiles and other emotional expressions are more frequent, stable, and enduring during REM sleep than during other behavioral states (Dondi et al., 2007; Messinger and Fogel, 2007).

Interestingly from an affective neuro-ethological viewpoint, only certain emotional dispositions are overexpressed in dreams, such as surprise, anger, fear, euphoric expectancy (SEEKING), or sexual desire, while, sadness, guilt or depressed affects are rare (Fosse et al., 2001; Smith et al., 2004; Nir and Tononi, 2010). Moreover, children preschool dream reports are often characterized by an explicit fulfillment of a wish (Colace, 2010, 2013). This fact confirms the original psychoanalytic view that dreams often constitute a hallucinatory expression of an unfulfilled desire, as well as our neuro-ethological view that imagination and dreaming are the expression of an introverted SEEKING drive. However, we think that this evidence does not necessarily confirm the Freudian assumption that dreams serve to discharge an excitatory somatic overload (drive-reduction theory; see next paragraph).

In adults, dreams are also characterized by reduced attention and voluntary control, delusional ideation, disorientation, impaired working memory, and lack of usual logical and linear spatio-temporal relations (Nir and Tononi, 2010). In an old but efficacious psychodynamic language, we can say that dreaming consciousness is characterized by an “abbaissament du niveau mental,” a collapse of the psychological tension that usually integrates mental contents and organizes them in a rational structure (Janet, 1889).

However, the studies on the ontogenesis of dreaming have shown that children preschool dreams (3–5 years) are frequently simple and without bizarreness (Colace et al., 1993; Colace, 2010, 2012). These evidences indicate that bizarreness is not an intrinsic property of neurophysiological events in the REM phase of sleep. In our opinion, the increase in dream bizarreness during development may be related to the increasing complexity of the individual brainmind and not just to the attempt to bypass repression, as suggested by Sigmund Freud. Indeed, the more the noetic memory system becomes ramified and differentiated, the more the dream will manifestly show those highly complex synthetic formations, which we call images, and which Freud though were a product of a condensation process. By doing so, the dream functions as a producer of virtual scenes that express the coherent and symbolic meaning of our inner world, which does not follow the rules of the external reality (Wilkinson, 2006; Alcaro and Panksepp, 2014). As suggested by Jung, “dreams do not deceive, they do not lie, they do not distort or disguise, but naively announce what they are and what they mean. […] they are invariably seeking to express something that the ego does not know and does not understand” (Jung, 1946, para. 189).

## On the Function of Dreams

In the attempt to ascertain the adaptive function deserved by the evolution of dreaming, the majority of research and theories focused on its role in the consolidation of memories (for a review, see Diekelmann and Born, 2010). These theories start with the phenomenological observation that dreams often incorporate experiences that the subject had during the day or within the days before Foulkes and Rechtschaffen (1964), Foulkes (1985) and Foulkes et al. (1989). It has also been demonstrated that the amount of time spent in the REM phase during the night following a training session has a positive influence on the person performance in the following days (Grieser et al., 1972; Barker, 1977). On the other hand, the selective deprivation of the REM phase has a negative influence on performance on the days following the training (Chernick, 1972). Further experimental studies in humans and animals showed that during the REM phase one witnesses a reactivation of the very same neurons and neural circuits that were particularly active during wakefulness (Pavlides and Winson, 1989; Maquet et al., 2000). Moreover, REM sleep is able to strengthening memory consolidation by activating the expression of genes linked to synaptic plasticity (Ribeiro and Nicolelis, 2004; Rossi, 2004).

It has been suggested that dreams are involved in the reactivation of cerebral circuits stimulated during wakefulness in a way that reinforces the new connections (Stickgold, 1998; Stickgold et al., 2000). Withdrawing oneself from the outside work, the organism sinks within a psychobiological “off-line” state that allows it to enter in contact with the recently acquired experiences, re-elaborating and integrating them with those already existing (Gais et al., 2000; Maquet, 2001; Mednick et al., 2003).

Although supported by an abundance of data, mnestic consolidation theories of dream’s function are not able to explain why dreams are not limited to repeating already-lived experiences, but instead they re-elaborate them in new and creative ways. A careful look into the contents of dreams reveals that they combine information acquired the day before with past memories as well with expectations and phantasies (see next paragraph). As suggested by Ribeiro and Nicolelis (2004) dreams “are hyper-associative strings of fragmented memories that simulates past events and future expectations” (p. 12).

The most influential simulation theory of dream’s function was proposed by the psychologist and philosopher Revonsuo. He sustained that dreams allow an off-line simulation of threatening events and promote the development and maintenance of threat-avoidance skills during wakefulness (for a recent review, see Valli and Revonsuo, 2009). It was further showed that such anticipatory off-line training of the brainmind was not exclusively directed toward avoiding aversive experience, but also approaching appetitive situations and conditions (for a review, see Perogamvros and Schwartz, 2012).

## The Primary and the Secondary Process of Thinking

The spontaneous and free-floating flux of imagines that characterizes dreaming and mind wandering has been recognized in psychoanalysis long time ago. Sigmund Freud referred to it as the “primary process of thinking,” described as the instinctual, visual and unstructured mental processing typical in children, in dreams, and in those with psychoses. Whereas for Freud the primary process is involved only the discharge activity of psychosomatic drives, for Jung it actually constituted a specific way of “thinking.” According to Jung, such processes carry not only an unknowable somatic aspect (Freud’s drives), nor just a fundamental affective content, but also a cognitive activity which may be potentially extremely developed, albeit functioning in its specific imagistic, metaphorical, condensed way.

In accordance with Jungian view, recent neuro-dynamic models suggest that dreaming and mind wandering are inferential predictive processes of the brain, where virtual realities are created in order to optimize organism’s expectations and performances within the external reality (Hobson and Friston, 2012; Moutoussis et al., 2014). Indeed, experimental data showed that REM sleep enhance cognitive flexibility (Walker et al., 2002), decision making (Pace-Schott et al., 2012), moral reasoning (Killgore et al., 2007), insight (Wagner et al., 2004), and creativity (Home, 1988; Cai et al., 2009; Schacter et al., 2012).

It seems then very plausible that the primary process of thinking permits a “psychic rehearsal of possibilities” (Ribeiro and Nicolelis, 2004) that unfolds a prospective adaptive function, helping to anticipate possible future events or to find solutions to unresolved problems or conflicts. Such idea was anticipated that C. G. Jung, who was in overt conflict with the Freud in assuming that dreams are “an anticipation in the unconscious of future conscious achievements, something like a preliminary exercise or sketch, or a plan roughed out in advance. Its symbolic content sometimes outlines the solution of a conflict…” (Jung, 1947/1954, p. 493).

In waking modern adults, the primary process is usually abandoned in favor of a more mature, linear, constrained and “rational” way of thinking, that we may call, after Freud, the “secondary process of thinking” and that is related to the emergence of a stable sense of self and of the external reality. Freud and Jung argued that the primary process of thinking is dominant in all unconscious ways of functioning, in human infancy and in the “savage” (sic!) man. However, healthy adults of our modern society it generally shows a new and more mature form of cognition, characterized by the ability to form metacognition and to self-reflection (Shimamura, 2000; Fleming et al., 2012).

It is very hard to describe what makes this fundamental evolutionary passage possible, and any attempt in such direction is beyond the scope of this contribution (see Carhart-Harris and Friston, 2010; Northoff et al., 2010b). However, following a neuro-psychoanalytic path, we may consider that in humans the attachment relationship becomes the instrument for an additional and fundamental evolutionary step in subjective mental life. This step has been described with the concept of “object cathexis” (Northoff, 2011), that is to say with the development from a spontaneous imaginative activity centered on affects to an imaginative activity centered on the representation of internal objects isomorphic to the external ones perceived^6^. This process is directly linked to the permanence and stabilization of object’s representations and self-representation, and permits the passage from the primary to the secondary process of thinking (Carhart-Harris and Friston, 2010; Northoff, 2011).

However, the primary process does not disappear, but it continues to live in the underworld in the form of unconscious fantasies that influence our mental processes, our moral evaluations, our behavior and, pervasively, our conscious activity. Moreover, the primary process of thinking may be consciously accessible in non-ordinary states of consciousness, such as dreaming, free-association, meditation, divergent thinking/creativity, sensory deprivation, near death experiences, psychedelic states, magical thinking, religious experiences, the onset-phase of psychosis and the dreamy-state of temporal lobe epilepsy (Carhart-Harris and Friston, 2010; Carhart-Harris et al., 2014).

The archaic mind-wandering function does not disappear in humans, but unfolds an essential *dispositional* re-equilibrating” function that permits to maintain the organism’s neuro-psychic identity and stability. Jung called it the “compensatory” function (Jung, 1935a, 1956). A quite similar hypothesis has been proposed by Jouvet (1975), who considered dreams a form of neurogenetic re-elaboration where new experiences are assimilated in accordance with the organism’s instinctual psychobiological nature. According to Jouvet, dreams constitute a form of neuro-genetic programming, where lived experience of contact with the external reality is assimilated and re-organized beginning with one’s own psychobiological identity. In our view, such process of continual re-programming theorized by Jouvet and Jung has a primarily affective origin. In fact, while during active wakefulness emotional dispositions are strongly tied to the conditions of the external environment, during imagination and dreaming they can be expressed more freely, influencing our mental activity in a manner that is direct and all-embracing (Hopkins, 2016).

## The Neuro-Evolution of the Imaginative Function

The evolution of the imaginative function follows a path that may be divided in three stages:

1. REM-sleep dreaming that is present in reptiles and constitutes the first form of mental imaging.
2. Mind-wandering that is present in endotherms and constitutes the amplification of a dreamlike imaginative function in the resting wakefulness.
3. Constrained secondary-process imagination that is present in humans and is characterized the by a stable sense of the Self and of the external (physical and social) reality.

### Reptiles and the Dreaming Brain

The evolution of terrestrial vertebrates is characterized by increasing complexity and plasticity in their neuro-psycho-behavioral organization and by the emergence of a new form of “noetic” consciousness that superimposes the core-Self anoetic awareness (Fabbro et al., 2015). Noetic knowledge refers to experiences that “arise when refined attentional capacities permit a clear distinction and categorization of specific features of the environment which, with enough neo-cortex, allows animals to think ahead. Indeed, when specific aspects of events become the focus of attention, explicit object-related reflective awareness comes into the fore while semantic (conceptual) memory helps to analyze and categorize the situation (Tulving, 1985). This is the form of consciousness that Edelman called “primary consciousness, ” and that he related to the activity of re-entrant thalamo-cortical brain circuitries (Edelman, 1989). Indirect evidence suggest that noetic consciousness is present across mammals, birds, and perhaps also in reptiles (Edelman et al., 2005), and that human babies, born very immature, start to manifest it after the 3rd month of development” (Alcaro et al., 2017, p.10).

As suggested by Jung and Jouvet (see previous paragraph), the evolution of noetic knowledge may threaten the maintenance of an integrated and coherent psychobiological identity (the Self). Therefore, the focused attention processes used by terrestrial vertebrates when they are dealing with the external environment may be compensated by the return to a “primary-process” unconstrained arousal that turns things upside-down. In such a way, the impact of the external reality on the organism is assisted by an inverse movement, through which the organism re-organizes its experience using an instinctual code.

Interestingly, recent evidences showed that, together with their novel noetic abilities, reptiles exhibit neurophysiological correlates of the REM sleep (Shein-Idelson et al., 2010), suggesting that a primordial form of dreaming evolved early in terrestrial vertebrates, well before the evolution of endothermic species (mammals and birds). Such kind of mental nomadism may be related to the off-line expression of a SEEKING-self-projective drive within the medial cortex, which constitute the reptilian antecedent of the MTL of mammals (Reiter et al., 2017). In producing an enormous amount of mental images and representations, dream imagination “help to solidify the many unconscious habits that are the very foundation of our personality. In the final accounting, dreams may construct the powerful subconscious or preconscious affective psychological patterns that make us…the people that we are. They may help construct the many emotional myths and beliefs around which our lives revolve” (Panksepp, 1998b, p. 142). In this essential mythopoietic process, dreams are the first ring of the reflective function, permitting that the “Self comes into mind” (Damasio, 2010).

### Endotherms and the Amplification of the Imaginative Function

In contrast to cold-bloodied terrestrial vertebrates^7^, mammals and birds present metabolic modifications that facilitate oxygen uptake, transport and delivery^8^ (for a review, see Hillenius and Ruben, 2004). Due to elevated rates of cellular oxygen consumption, mammals and birds are able to sustain higher levels of activity for extended periods of time, with considerable ecological advantages. Such stamina enables these animals to forage over large territories, and/or to perform extensive seasonal migrations in pursuit of favorable resource conditions (Hillenius and Ruben, 2004).

The increased cardio-pulmonary capacity not only affects behavioral activity, but also potentiates *resting metabolic rates*, that in mammals and birds are 10–15 times greater than in reptiles (Bennett, 1973; Schmidt-Nielsen, 1984). The increased resting metabolic rate allows for increase body heat and for regulating it in an autonomous mode, independent of the external physical environment (Hillenius and Ruben, 2004). Therefore, endothermic species can use the excess metabolic capacity to increase internal order, homeostasis and their living subjective autonomy (Torday and Miller, 2018).

The increase in metabolism does not influence only body heat, but effects also the level of resting-state brain activity. Although ectoterms show a consistent reduction of brain metabolism when they are not engaged within specific actions, mammals and birds periodically show brain metabolism during sleep and resting wakefulness that reaches and even exceeds the levels typical of periods of active engagement (Mantini et al., 2011; Lu et al., 2012; Raichle, 2015). Therefore, in endotherms, the endogenous self-referential inferential processing is not restricted, as in reptiles, to brief off-line REM-like arousal states, but occupies wide portions of sleep and resting wakefulness. In such a way, they are able of greater exploration (SEEKING) not only directed towards the external world, but also towards the mental internally-generated world. Such important amplification is related to the evolution of some ventral and dorsal midline cortical brain structures (Raichle, 2015).

In our neuro-ethological view, the imagination of endotherms is closely related to the evolution of complex socio-affective relational patterns that may be modified by experience and constantly re-programmed within resting-state DMN activity. In accordance with such hypothesis, the neuroanatomy of their social emotional systems (PLAY, CARE and PANIC/SADNESS) extends to cortico-limbic midline areas belonging to the DMN^9^ (Panksepp and Biven, 2012). It seems also plausible that in the early infancy, the attachment process, sustained by basic emotional drives, is open to modulations exerted by the imaginative function.

As a consequence of their amplified imaginative function, endothermic species show complex social, cognitive and affective capacity, such as creative thinking, the ability to fake, some form of a “theory of mind,” etc. (Griffin, 1985; Crystal, 2012; Roberts, 2012). In particular, the inferential and innovative aspect of the resting-state imaginative activity influences the communication skills by enriching them with a repertoire of potentially unlimited signals, signs and symbols^10^. An illuminating example in this regard is that of bird singing in migrating birds such as the *Sylvia atricapilla* (Portmann, 1986), in which repetition and creative spontaneity are combined, giving birth to a unique and unrepeatable language.

Interestingly, recent evidences show that birdsong sleep is characterized by periods of neural activity implicated in song-replay (Derégnaucourt et al., 2005), that unfold an important function in the vocal learning of songbirds (Shank and Margoliash, 2009; Margoliash, 2010). Although the contribute played by different sleep stages in such a process has not been determined yet, experimental data show that the plasticity of vocal songs is incremented by certain oscillatory bursts of neural activity that in mammals accompanies REM sleep and the activation of the SEEKING brain system (Yanagihara and Hessler, 2012).

### Humans and the Evolution of Verbal Thinking

Originally, the imaginative function was essentially an “off-line” process that takes place in REM sleep (reptiles). With the evolution of endotherms, the DMN resting-state patterns is attuned in a “transitional space” where internally generated fantasies and external communicative actions overlap. In the human species, such transitional space realizes its vast potential due to the evolution of linguistic communication, a tremendous jump that separated our species from other animals and that is the base of all forms of human civilization.

With the acquisition of language, the flux of visuo-spatial representations may acquire the structure of audio-verbal thoughts. In such a way, during human development, the collective and transpersonal dimension may be interiorized within individual psyche, permitting the kind of cultural revolution that is linked to and completes biological development.

It has been supposed that the evolution of language permitted the development of “autonoetic consciousness” (Fabbro et al., 2015), defined as the capacity to represent themselves across space and time, and to build a stable and conscious psychological identity, that we may call the “ego.” For example, Sigmund Freud underlined how verbal thinking is the main form of self-conscious perception of internal mental activity. The importance of verbal language and specialized linguistic brain areas for self-consciousness has today been confirmed by influential neuroscientific theories (Edelman, 1992).

However, the psychological analysis of patients with cerebral lesions shows that damages to the right hemisphere induce deeper personality diseases and deficit in self-consciousness than equivalent lesions to the left hemisphere, where language areas are located (Kaplan-Solms and Solms, 2000). For example, not only right-damaged patients tend to completely neglect their diseases (anosognosia), but they often present severe psychiatric symptoms, such as profound melancholia, paranoid ideation or a complete collapse of the ego functions as observed in florid psychotic states. Kaplan-Solms and Solms (2000) concluded that the right cortico-limbic network is implicated in “object catexis,” the process through which mind wandering is constrained by experience-dependent operative models of attachment organized within the right brain (Schore, 1994). This kind of containment is necessary to the passage from the primary to the secondary process of thinking, and then for a stabilization of ego and objects representations (Carhart-Harris and Friston, 2010; Northoff, 2011).

A way to integrate the linguistic and non-linguistic theories of autonoetic consciousness is to suppose that verbal thinking permits the passage to the secondary process and amplifies its functioning only when the subject is able to distinguish the internal from the external world. This differentiation presupposes the mentalization of the experience of being separated from the desired/loved object (Klein, 1921; Bowlby, 1969/1982). In our neuro-ethological perspective, it means the possibility of a complete expression of the PANIC/SADNESS SEPARATION DISTRESS emotional complex within the conscious self-referential imaginative activity. Only in such case, verbal thinking and mental imaging may be properly attributed to an internal psychological space, which is different from the external social world of others^11^.

We are aware that this is a very important theme, which needs a deeper and wider analysis, especially in consideration of the process of “object catexis” (Northoff, 2011). It is then our proposal to dedicate to such argument an entire article that will complete a trilogy about the neuro-evolutionary architecture of human mind. Such trilogy started with the article about the affective core of the self (Alcaro et al., 2017), continued with this article on the primary-process imaginative function and will finish with a work on “object catexis” and the secondary process of thinking.

## Psychodynamic Considerations and Psychotherapeutic Applications

In the following pages, we will try to sketch our idea of the psychotherapeutic process (which fundamentally refers to the paradigm of Analytical Psychology) on the basis of the considerations that we have presented in the previous pages. In order to do so, we must once again recall our view of the mindbrain as a dispositional organic whole, which is activated “bottom up” and “inside out.” This means that the origin and the foundation of the mindbrain’s activity is activated from anoetic anobjectual affective states through a noetic endogenous imaginal production (image schemas), towards a autonoetic-verbal domain. All this is coherent with Jung’s idea that the primary level of mental processes is fundamentally *affective*. This first pre-formed affective motivational layer (the readiness to respond) acquires a complete psychological shape when is connected to—represented by—adequate “images,” that actually act as gestalt-patterning structures (Bucci, 1997). As we said, this connection between affects and images is a fundamental evolutionary gain (in endothermic animals) and is the precondition for a healthy mental development in ontogenesis. Indeed, without the adequate imaginal containment, the affective states of the Self have a diffuse and undifferentiated presence and are therefore projected directly onto the external perceptual world. In such a way, these indistinct emotional states and the objects of the perceived external world overlap. In other words, the individual is not able to “reflect on experience” and to distinguish the internal and the external.

If, as Jung maintained, the unconscious is formed by complexes of representations, which give shape and express fundamental affective dispositions, then producing appropriate images means to re-structure the integrative function in order to interpret and then respond to stimuli (i.e., to experience) in accordance to the nature of the “core-Self.” Jung described this imaginative process in his *Red Book* (Jung, 2012) or in his essay on the so-called “transcendent function” (Jung, 1916/1957). This process is experienced by artists (Carta, 2017b), who are able to first (receptively) *find* and then (actively) *give* an imaginal shape to their feeling states.

For Jung, the two fundamental ways to access the unconscious formations—what he called “complexes”—were dreams and the emergent imagery activated through “active imagination,” both of which possess a noetic form of consciousness. Both dreams and active imagination may eventually be followed by an autonoetic process through which the emergent noetic images may be analyzed and verbally shared in an autonoetic (self-reflectively conscious) state.

As we said, the noetic activity is the core of “active imagination” (Jung, 1916/1957, 2012), a process analogous to D.W. Winnicott’s concept of *non-integration—*i.e., of a relaxed/empty state of mind in which the ego may come into contact with endogenous impulses of the “True Self” (Winnicott, 1964, 1965). It is also similar to Bollas’ concept of the *unthought known* (Bollas, 1987), which refers to an archaic objectless affective activation that, in some “regressed” patients, appears like a form of diffuse mood. Active imagination has also been described at length by Khan (1983) as a state in which the mind “lies fallow,” i.e., retires from activity in the outside world in order to restore its connection with the Self. Such a state is connected with the capacity to be alone, i.e., to withstand a hollow resting state open to the reception of inner stimuli (images). This state may be also connected to the crucial moments of *alert inactivity* (Wolff, 1966), during which the newborn probably rehearses and explores through images new associative links in order to give emotional stability and cognitive coherence to his experiences.

From all we have said up to this point, we are now in the position to sketch some fundamental principles for a psychotherapeutic approach, which are based on the process through which the dispositional brainmind forms its *endogenous images*^12^ (hence not necessarily perceptions, nor memories of perceptual biographical events). This implies the departing from experiences/representations of outer/perceived objects towards a state from which endogenous, conceived images may arise. Therefore, in a way anti-behavioristically, we will not work on directly coping with “outer” facts, but we will *primarily* look for “inner images,” therefore restructuring the mindbrain capability to spontaneously form images. In a way: to learn how to properly *dream* in the waking state within the analytical situation.

We stress the word “primarily,” because it is obvious that the relationship between the inner and the outer environment—the brain-mind and the outer world—is a circular one. What we are trying to say may be expressed by comparing, in a somehow stretched manner, the main orientation of behavioral psychotherapy (BT), and of a noticeable part of cognitive-behavioral psychotherapy (CBT), with our model. It seems to us that, *within the circular process between disposition and situation—*between the brainmind and the environment—BT and CBT focus their attention on the outer object (on the situation). For instance, in order to cope with a phobic response, BT or CBT may systematically intervene on the way the subject deals with the perceived object by trying to better respond and adapt him to the situation. If the phobic object is a mouse, or an elevator, the psychotherapist will focus on them.

Our aim is apparently the same—to provide a more harmonic adaptation to the environment. Nevertheless, we would not consider the perceived object, which has become so salient for the phobic patient, as the true object that the patient is attracted to, but as a “fetish” that contains, or refers to, another, unconscious self-referential process that the subject is not yet able to imagine for a number of reasons. Such unconscious process is activated by an anoetic affect, which is projected onto the outer object (the phobic, fetish) *in search for an imaginal* shape^13^. In a “normal” situation, outer objects may provide a good-enough imaginal shape to the subject’s anoetic objectless affects, so that we may normally not be aware of the fact that, in truth, every meaningful (affectively salient) relationship represents the emergence of an inner, endogenous, potential affective image. As Winnicott explains, the shape that the outer object lends to the endogenous affect *should* be “just” good enough, in order to promote and maintain the activity of the SEEKING system at a productive level, and hence foster creative adaptation^14^. Therefore, our work will not be directed towards the outer—perceived—object, but towards the autonomous image that we expect will raise from the patient’s unconscious *via* the anxious, phobic feeling tone that the fetish contains. Once again, the process is actually circular, but our approach does not aim at adapting the subject to his situation, but to help him to dispositionally interpret it as something that is at the same time “found and created” (Winnicott, 1967)—i.e., transform facts into meanings. In order to do so, we must help the patient to look at his objects as projected images which have a symptomatic, and not a symbolic, character, as they are not yet able to express the patient’s dispositional affective nature within his environment. In other words, such object-relations express an inability not just to adapt, but to adapt in a personalized (creative) way to the world, because, as Heidegger would say, humans do not live in an environment, but into a *world*.

More generally, we usually do not interpret anxiety as a non-specific response to an environmental menace, but as the failure by the brainmind to form “good images,” i.e., images that, following Jung’s model, may give an imaginal noetic shape to affects that organize the Self into complexes and, eventually, into an autonoetic, verbal activity. Thus, we think that the psychotherapeutic efforts to directly deal to the outer object-world, especially through higher cognitive functions, actually point to a secondary level—a level of reaction/adaptation. On the contrary, our model aims to focus on a primary level that deals with the failure of the brainmind to form good images, i.e., to properly organize its own bottom-up activity, from basic raw affects to self-reflective consciousness, through spontaneous endogenous noetic image-formation process.

In our perspective, the spontaneous formation of feeling-toned conscious images is therapeutic *per se*, since it creates the conditions where rejected affects (this rejection may reach the level of what Lacan, (1958) called “forclusion”) may gain access to the imaginative field and then may be eventually perceived by self-awareness. This is possible by the creation of a condition for a possible regression towards a state of relaxation, or non-integration, that favors spontaneous imagination. Such a regressive condition has to do with the “regressive” shift from autonoetic (verbal) consciousness towards noetic, pre-reflective awareness. Jung (1946) theorized in length on this condition, but also Freud had this in mind when he wrote about a regressed state of the patient laying on the couch, in which the patient can associate “freely” while the analyst maintains a free-floating attentive state. Both these two authors make it clear a fundamental condition for psychotherapy: not only the patient, but *also the psychotherapist must maintain a noetic level of consciousness*.

Being fundamentally affective, the patient’s and the analyst’s endogenous images will be representations of their inter-affective, analytical field: their psychological *place of meeting* (see Stern, 1985; Sander, 2007) often described as *transference*. The creation of this transferential field is made possible by the analyst’s disposition to affectively/imaginatively tune in with the patient. In doing so, the analyst must be open to any signal that belongs to what Jung, on the basis of his theory of (psychosomatic) complexes, called somatic transference. Within this state not only verbal meanings are important. What is actually central are expressions which are based on *implicit* meanings, and which may be conveyed by non-verbal expressions, or by specific *actions*^15^. By this the therapeutic relationship may gradually turn into a sophisticated form of reciprocal *active imagination*, in which the analytical couple forms a relational network formed by the combination of their conscious and unconscious processes (for a description of this situation, in which the analytical relationship is also a co-imaginative process, see Jung, 1935b^16^).

It is crucial that, within the transferential field, the analyst sinks into a double mental state in which he is at the same time self-reflective and in contact with his inner noetic (dreamlike) activity. The therapist must be able to master a *double* state of consciousness—a noetic form of imagination monitored by an autonoetic activity—a hybrid state necessary in order for to perform what Bion (1985) called *rêverie*^17^. This implies that he will be tuned with the patient’s psychological formal activation level, so that the images that rise in the therapist’s mind, inter-affectively tuned with the patient may actually be (also) the latter one’. One of us has discussed this situation, and called it “ecstatic” (Carta, 2017a). It will happen that, in this state, images will flow through the analyst mind, too. They should be taken into close and critical account, as they may be aspects of the patient’s anoetic (purely bodily/affective) states that are projected into the analyst, or that the analyst just feels. The same may be true for elements of the analyst’s somatic transference.

Thanks to the double-state of its mental activity (noetic and autonoetic), the therapist is in the position to verbally reflect on these images in an autonoetic conscious state. For a patient this means to be able to express the noetic emergent images and to negotiate them with the analyst—an exceedingly important function, indeed. If the image is the reflection of the affective state upon itself, the autonoetic word is the self-reflection of the image. Nevertheless, during the imaginative noetic phase the verbal nature of the cure must be suspended. First, let the images flow, and then talk from them about them.

We would like to stress that the transformation from image to word is gradual. In fact, before words may fully express self-reflectivity and a mature object relatedness (with no confusion nor split between self and object), the quality of the language is deeply metaphorical. In fact, metaphors are the verbal formations closest to images, as they tend to condense two terms into a hybrid third one, which, like images, will be understood through a synthetic apprehension, *en masse*. At this stage of the process, non-metaphorical, intellectual, analytical words may feel irrelevant, abstract or, worse, dissociating due to their intellectual/ intellectualizing nature. In our experience, metaphorical language is by far the most frequent form of language that flows between patient and analyst. It is a non-yet fully conscious (autonoetic) language, but it is the direct verbal immediate product of the transformation of affective contents expressed or expressible through images (dreamable).

In sum, the function of the psychotherapist and the therapeutic process may be recapitulated as such:

1. **Description**. The psychotherapist helps the patient to describe his situation. He will not try to intervene on the patient’s representation of his extroverted objects, but will help him/her to focus on the feelings and on any association that may arise from this representation. If, for instance, the patient will refer to his/her phobia of flying, at this stage the psychotherapist will not suggest any interventions or any possible modification, as the image of flying may not actually be the best image connected to the original affect, but just a second associative carrier for the affect itself. We think that from this stage, the language spoken is already deeply metaphorical. Intellectualizing, theorizing, or sharp analyses are now counterproductive. The psychotherapist must *already be in a double state of consciousness*.
2. **Focalization. Patient sinking into the resting state**. At this point, the psychotherapist helps the patient to depart from his/her involvement with extroverted images/objects, and focus on his feelings. In fact, in order to let the endogenous image raise it is imperative that the patient attentively focuses on the feeling-tone, or the specific mood, which is the *prima materia* out of which the images are formed. Such a focus must also be directed towards bodily feelings, tensions, movements of any kind, while a correct breathing is secured^18^. While the patient sinks into the resting state and begins to imagine, he should not speak. Verbal activation and speech may actually interfere with the process of imagination.
3. **Containment**. The psychotherapist must contain the patient as he/she experiences contact with endogenous images. This means to be able to *go where the patient is*, which we believe to be the cornerstone of psychotherapy. This process implies a mature and sustained empathy, and the ability to somehow see *who the patient is, and in which emotional/imaginal environment he/she really is*. Such containing function is important also because the patient’s enteroceptive and proprioceptive experience may be somehow modified during the emergence of his/her feeling-toned endogenous images. His body schema and his body image (Dolto, 1984), hence his/her feeling of his/her subjectivity me be perceived as if he/she is, for instance, a certain kind of child.
4. **Negotiation**. The psychotherapist must help the patient to interact and negotiate with his/her endogenous images. During this stage the patient will still limit his/her verbal interactions to just briefly report, should this be needed, what is happening between him/her and his/her images.
5. **Elaboration**. At this stage, the patient may share his/her experience with the endogenous feeling-toned images and the interactions he/she had with them. During such phase, endogenous images will be matched with the patient’s “real,” objectual life experiences that he/she reported during phase (1) and with associations with further objectual life experiences and with past memories. Here, the psychotherapist experience in mastering his own dual state of consciousness will be his/her fundamental tool to go back and forth between these two realities, and transfer the emotional-cognitive-relational meaning of the autonomous images into the patient’s life. At this stage, the language used may become less metaphorical, more analytical and, somehow, abstract, as it may autonoetically express and shed light on the relationship, the links-between.

Before presenting a very brief example of this process, we are in the position to clarify that its bottom-up nature does not actually describe it in a fair way. In fact, the psychotherapeutic process we are describing* begins* at the “bottom”—affects and somatic activation—*via* images, through metaphors, towards autonoetic cognitive contents, but in its ongoing nature it actually describes a cycle, in which we find its necessary place for the up-down flow from cognitions towards affects, as in phase (5). Yet, we want to point out again that this last (autonoetic, verbal) phase may actually not occur, and that the re-integration between the affective/motivational/intentional layer and the patient’s experience with his relational reality may take place *implicitly*. In fact, we think that this cycle is constantly happening implicitly at every moment of one’s life. Our description within a psychotherapeutic setting is just an attempt to describe it and show how it may be “used” in a psychotherapeutic relationship.

## A Clinical Sketch

What follows is a very condensed clinical sketch. Our purpose is not to discuss a clinical case (as this sketch is not even a clinical vignette), but to show a possible way to apply what we have been discussing so far in the clinical domain.

The patient is an unmarried, 40-year-old, man. He is going through a period of pervasive distimic mood, in which he is gripped by feelings of usefulness, desperation and passivity. He is stuck in his relational world (both with his relationship to his girlfriend, which he cannot transform into a stable commitment which could transform him into a father, but also with his colleagues) and in his professional/creative area, as he does not feel able to be intelligent and productive. During the previous session, he was feeling in a kind of swamp (this is an image). He described, once again, what had happened between the sessions, in relation to this depressive feeling (DESCRIPTION).

At the end of the hour, he suddenly remembered an old dream. The therapist told him that they could go back to that dream in their next session, which is the one we are describing now.

P. I searched among my writings for a description of the dream that came up at the end of the last session, but I could not find it. Nevertheless, I remember the facade of a small Baroque style church that is set in light-colored rock. I enter through the door and am inside a large cavern, it seems like a uterus, dark and full of blood. I am entranced by this image. There is a naked woman, crucified on two large, rough tree trunks that have only been thinned of their branches, which hang up high near one of the walls. Above me a witch flies round and round and I am tied to a large trunk that is laid on the ground, on one side by my feet and the other by my arms which are stretched over my head. I am naked and the witch swoops towards me in a nosedive while the crucified woman thrashes about weeping and crying out in pain.

This dream is from many years ago and it makes me think of how much time has passed and how these themes are still so much alive in me. What has changed, in your opinion in these years?

T. I think in some things you have changed and in others less so. You still do not devote yourself to engage in your being in the world and you do not commit thoroughly to your relationship with others, especially in the case of your girlfriend.

There are a few minutes of silence.

P. In the past few days I have felt very tired. I have trouble getting up in the morning and I have no energy. Saturday I have to give a seminar and the thought of it crushes me. These days I feel very tired. It is difficult to get up in the morning. I have no energy. Saturday I have to hold a seminar, and this thought crushes me. (Here, the phase of DESCRIPTION seems complete).

T. What image do you have of this?

P. There is no image. I am just afraid to remain speechless, to make a mistake, to get stuck, to find myself in a deadlock.

T. What feelings come up when you speak about it?

P. I do not feel anything, I just feel very tired.

T. You should try to stay with this feeling tired, and try to let image emerge. (FOCALIZATION).

A. few minutes pass as the P. sits with his eyes closed. He knows he should not talk while he imagines.

P. I pictured a swamp with mangrove trees, I see the bottom of the swamp of dark-gray mud under a half meter of clear water as if it is a lateral cross-section. I see myself, naked, sinking into the very dense mud, I am tired, exhausted, I see my feet that as they are moving, sink. I tried to get out, I attempt to walk in the low water of this endless forest. Perhaps, though, this imagining of getting out corresponds to not wanting to stay in this emotional situation. (FOCALIZATION).

T. You interrupted you experience of imagining. Can you resume it? (CONTAINMENT).

Several minutes pass.

P. I imagined myself to be in the deep sea (phrase also means “in trouble”), floating naked and trying to hold on. I felt lost, desperate, and that I will soon be dead. I have looked all around me without seeing absolutely anything. The sea has become dark and grown rough. I am forced to float straight up. I was always more exhausted and desperate.

T. You did not do anything at all to say yourself…

The P. comes to a standstill. He says “What could I have done? There was nothing that could be done.” Nonetheless the non-verbal cues by which he expressed himself, sounded to the T. as a defense. It sounded like an intellectual defense, a distancing into the autonoetic layer of the noetic imaginative process linked to affects.

T. Are you sure there was absolutely nothing that could be done?

The affective-relational atmosphere generated by the strong therapeutic alliance, founded on solid trust, makes it possible for the patient to take the question seriously and it lets him to reconnect with the image. (CONTAINMENT).

P. Well, I looked around but I did not see people or other…

T. But you did not call for help, for example, perhaps someone or something would have heard you. Who knows what might have happened?

Minutes of silence pass, while the P. intertwines his hands and looks around, and T. for a moment feels deeply sleepy. (CONTAINMENT. The T’s sleepiness is a sign of his projective identification with the patient’s unconscious, dissociated affects).

After several minutes of silence which make the relational field intensely concentrated, something moves in P., his face relaxes and his body assumes a more open position. T. feels that P. has understood something.

P. In this moment, some imagines came to mind of a film I saw several nights ago. The film is called “The Grey” and tells the story of an airplane that crashes in Alaska. The protagonist Liam Neeson guides some of the survivors towards possible safety from hypothermia and wolves. They start off with eight people but perhaps only he is able to save himself. In the most critical moments, the protagonist is able to access images and memories: he images that he is lying on a bed with his beloved and she says to him “Don’t be afraid” and he remembers being in his father’s study and reading a poem that has framed and hanging on the wall, that he recites like this “Once more into the fray, Into the last good fight I’ll ever know, Live or die on this day, Live or die on this day.” After the images of this film, I saw the image of my girlfriend’s mother, who is dying and is passive and angry at the world for what has happened to her, and then my mother who was angry with everyone for her unhappiness.

T. You imagined your Anima-guide and a paternal figure that help you in finding a solution, a way out of a dramatic situation, how do you feel now? (NEGOTIATION).

P. I feel to have re-surfaced (he pulls himself up on the seat of the chair).

A few seconds pass.

P. The feeling of fear returned, thinking of Saturday.

T. And then?

P. I imagined a solution to deal with it.

T. Allow the image to form itself. Let it go. (CONTAINMENT).

P. I imagined to be a warrior and I remembered a dream about Kung Fu that I had last week. I was in a Japanese house with three other people. A samurai woman with long, black hair tied back and a sort of light armor was accompanied by a second person, was coming towards me and I threw an object at her with an astounding gesture that I was amazed that I had actually performed. The woman dodged it without difficulty and looked at me as one would a master or a very capable warrior. (FOCALIZATION).

Using a Jungian metaphor, the T. thinks that the P. had restored the image of a strong, determined and capable Anima in place of a wounded and passive one. (ELABORATION).

T. How do you feel now?

The P. confirms what had already clearly been expressed by his posture (now more vigorous) and my his facial expression, more awake and alert.

T. It seems that now you can decide to take an action, and you can truly do it. (ELABORATION).

P. Yes, it is really like that^19^.

## Author Contributions

AA had the original idea and developed mainly the neuro-ethological part. SC introduced important theoretical and clinical contribution, especially in relation to Jungian perspective and image formation.

## Conflict of Interest Statement

The authors declare that the research was conducted in the absence of any commercial or financial relationships that could be construed as a potential conflict of interest.

## Footnotes

^1^Attention usually shift back and forth between the external and the internal world, and this correspond to parallel shift between the DAN and the DMN. In addition it has been identified a right-lateralized ventral attention network (VAN) which is activated by salient stimuli and re-direct activity from internal-oriented DMN activity towards the DAN (Corbetta et al., 2008).

^2^But what does “Self” mean? Although the “Self” may be viewed as the product of a self-conscious representation, it is also possible to define the Self on the bases of certain minimal essential characteristics that do not presuppose “autonoetic knowledge”: the feeling of agency, the ownership of feelings from the body, the experiencing the self as a unit, the labeling of stimuli as self-referential, etc‥ As suggested by Weiler and coll., this “minimal self is the most basic level of the self and refers to the consciousness of oneself as an immediate subject of experience that is temporally and spatially confined to the immediate present (Clare et al., 2011); this self does not require language nor working memory but only a short-term memory (Damasio, 1998). It has a relationship with time processing in the sense that perceptions and actions should be continually sensed by someone; disruptions in this system (and therefore in the “minimal self”) are commonly seen in schizophrenic patients (Martin et al., 2014; Weiler et al., 2016, p. 246).

^3^With “matching,” we are referring to Sander’s (2007) analysis of human interactions within a complex dynamic system theory point of view, and to Winnicott’s elegant paradigm of the object presenting (Winnicott, 1964, 1965).

^4^“Entropy” measures the unpredictability of the system, its degree of “apparent” randomness and distance from a recognizable order (Ben-Naim, 2008).

^5^It should be mentioned that it has also been recognized the presence of another source of constraints, different from automatic constraints. Deliberate constraints consist of cognitive evaluations and judgments that are explicitly directed towards a goal and that may be overtly represented within the ego consciousness. These deliberate constraints seem to be related to the activity of the dorsal subcomponent of the DMN, which include the dorsomedial prefrontal cortex, the lateral temporal cortex and the inferior frontal gyrus (Christoff et al., 2016).

^6^However, a failure in the postnatal attachment relationship may lead to a block of social communication and to variety of pathological outcomes as a consequence of the dissociation between core emotional feelings, internal autonomous imagery, and action through verbal and non-verbal expression.

^7^It has been shown that cold-bloodied terrestrial vertebrates show lower respiratory rates than endothermic vertebrates, and present a predominant anerobic metabolism (Bennett and Dawson, 1976; Bennett, 1991). Such low metabolic potentials of ectoterms reduce their ability for spatial exploration. Although they are able of bursts of intense exercise, they rapidly fatigue as a result of lactic acid accumulation.

^8^For example, endotherms show increased pulmonary capacity, ventilation rate increased blood volume and blood oxygen carrying capacities, fully segregated pulmonary and systemic circulations and expanded cardiac output, as well as increased mitochondrial density and enzymatic activities.

^9^It has been noted that three elements of behavior mark the transition from reptiles to early mammals (and birds): nursing, play, and the separation call (MacLean, 1985). Further studies in affective neuroscience have localized specific subcortical brain circuits responsible for such social behaviors and their characteristic emotional feelings: the CARE/nurturing love system, the PLAY/joy system and SEPARATION distress/panic-sadness system (Panksepp, 1998a; Colonnello et al., 2011; Panksepp and Biven, 2012; Panksepp and Panksepp, 2013). All three systems use vocal and non-vocal channels that initially develop during attachment and then are used in all other types of social interactions.

^10^Neuro-evolutionary studies performed by Stephen Porges have shown that emotional expression and communication (facial expression, sucking, swallowing, breathing, crying, vocalization, etc.) are controlled by cranial nerves and muscles that evolved from the primitive gill arches that extract oxygen from water. Moreover, they depend on the efficient functioning of the ventral vagal complex (VVC), a parasympathetic system that evolved in high vertebrates to regulate the internal state of the body in a flexible and rapid way (see Porges, 2011 for a compendium). Together this suggests a means of tuning the psycho-physiological regulatory function to socio-emotional communicative processes, as well as of registering the intersubjective social environment on the internal affective world of the individual (see Trevarthen and Aitken, 2001, for a review).

^11^We are aware that this is a very important theme, which needs a deeper and wider analysis, especially in consideration of the process of “object catexis” (Northoff, 2011). It is then our proposal to dedicate to such argument an entire article that will complete a trilogy about the neuro-evolutionary architecture of human mind. Such trilogy started with the article about the affective core of the self (Alcaro et al., 2017), continued with this article on the primary-process imaginative function and will finish with a work on “object catexis” and the secondary process of thinking.

^12^Of course, such images may acquire, through projection, the likeness of outer objects. This is clear in fetishism and phobias, in which the objective, perceived, outer object acquires a value-meaning from the projection of an endogenous, still unconscious affect-image image. Therefore, also in such cases we think that the “active principle” that produces a phobia or a fetish is to be found “inside” the object, which acts as a carrier. Hence, the best approach should not be from the outside towards the inside of the mind, or from perception to cognition and emotion, but “inside out”—the other way around.

^13^If we turn our view from the outer object, to the brain, we may refer to Panksepp’s (1998b) view of the role of the amygdala regarding FEAR. He maintains that the amygdala is not the origin of the FEAR affect, but that it is an associative site in which the original raw affects (the unconditioned stimulus) arising from lower regions of the brain, especially the PAG, are redirected within the brain and associated with conditioned stimuli.

^14^Without this character of non-perfect adaptation of the inner endogenous image to the environment, our specie would be extinct, as humans need plasticity in order to compensate their neotenic biological endowment.

^15^In this case, such actions are the opposite of acting outs, as they not only do not substitute meanings and block change, but actually convey an implicit, condensed complexual meaning that seems to be finding its bottom-up way from affects, through the noetic action, into a autonoetic, self-reflective process.

^16^If, as Jung says, in the wakeful state we are the subject of all objects, and, [‥.] in complete reversal of my ordinary consciousness,” in the unconscious I am the object of all subjects (Jung, 1959, para. 46; or in dreams, as in the dream every image possesses an autonomous subjectivity), in the noetic state of mind like that that supports active imagination, the affective images may autonomously flow into the analytical space, while the egos of analyst and patient still participate with their own specific subjectivity. In fact, within the individual, private imagining process the ego may imagine to interact or to talk to these images in a brainmind state that we think is based on the activity of the Default Mode Network Network (DMN). The same may happen in a relational space, in which the other is at the same time perceived and conceived. This “imagining the other” has obvious transferential connotations. Nevertheless, far from being always blind and narcissistically wish-fulfilling, it may actually grasp, through unconscious perception (intuition) elements of truth that are still unapproachable by autonoetic consciousness.

^17^An important distinction between our position and Bion’s rêverie, is that the contents of the latter are not just what he called “beta elements” (raw sense-data and of “inchoate elements,” Bion, 1967) but may be, due the imaginal nature of the noetic processes, also affective images, which corresponds to what Bion called “alpha elements.”

^18^There is no space here to underline how the somatic level, is crucial in order to lay the ground for the whole therapeutic process. Breathing, for instance, is a fundamental aspect of such a sinking towards the resting state from which images may rise from the patients’s feelings, or the mood the patient feels (Zelano et al., 2016; Piarulli et al., 2018).

^19^As we already wrote, we chose to avoid every interpretive aspect of our clinical sketch, which clearly involves a complex relational network between the patient and his endogenous mother complex, which shows evident impersonal, archetypal qualities, not totally deriving from his direct experience with his mother-in-the flesh. The patient’s mother complex seems responsible for the his feeling of being psychologically and energetically castrated—hence for his depression—together with his intense emotional difficulties involving his relational world.

## Appendix 1. The Formation of Image Schema

In cognitive psychology and neuroscience, images are the (re)presentational emergences of structural configurations, which are analogous to what are called *schemas* (Sheets-Johnstone, 2010; Cappuccio and Wheeler, 2012), or *maps*^20^ (Damasio, 1999, 2010), and, which a more engrossing and transversal meaning, Jung called *complexes*.

According to Mandler (1988, 1992, 2000), *image schemas* emerge from the comparison between stimuli and are the base of abstract concept formation. Mandler refers to *perceptual analysis* as the fundamental procedure through which, *using* external perceptions acquired by what she calls *perceptual recognition*, the brainmind expresses, at a mental level, the innermost symbolic sense of its life. For Mandler, perceptual recognition is a sensorimotor activity, which takes place automatically and does not require any conceptual framework. On the other side, perceptual analysis is an active process of comparison between stimuli, and is the earliest evidence of a contemplative attitude (Knox, 2004a) and constitutes the basis of abstract concept formation.

Along with Knox (2001, 2004a,b), we consider the mechanism of perceptual analysis as described by Mandler a persuasive model that may explain how mental images are formed. One fundamental point is that schematic images are not produced by “perceptual recognition.” In fact, it is the active, innate process of perceptual analysis that *uses* perceptual recognition *in order to form re-presentations of the intrinsic dispositions of the living organism (instincts, drives, emotions)*. In this sense, image-schemas may be equated with Jung’s archetypes-as-such, and their patterned representational content may be referred to what Jung described as the “self reflection of the instinct upon itself” (Jung, 1948, p. 257).

Here, we disagree with Fonagy (2002), for whom the early psychic structures are produced through (not modified by) early pre-verbal infant experience before the emergence of consciousness. Therefore, due to this situational tilt or self-environment interaction, for Fonagy any psychological imagery can be experienced as if it is innate, when it actually is not. Our dispositional tilt also contrasts with the views shared by Knox (2004a); Saunders and Skar (2001) or Merchant, who, reversing Jung’s psychogenetic theory, see archetypal imagery as an emergent phenomenon arising out of mind/brain structures (the image schemas) which have been laid down in early infant life as a result of the infant’s developmental experience (mostly interpreted through attachment theory), so that, while the complexes occur first, the “archetypes” would be a label arising from further categorization of these archaic experiences.

Consistent with the Jungian perspective, we must strongly emphasize that the isomorphism between the image-schemas and the object world to which the former refer does not mean that the self-produced mental images resemble those of the outside. Our starting point for our dispositional tilt is given by our extraordinary capacity, based on the “knowableness” of natural numbers on the part of the human species, to “know” the “external reality.” In fact, the reflective isomorphism that we are referring to is based on the radically “transitional” (Winnicottian) nature of natural numbers, which are simultaneously invented by the mind and found in the outside world. Therefore, the image-schemas we are referring to represent the “means” (rooted in the perceptive structures, for example visual, but not only), to reach, through a process of abstract refining, their ultimate mathematical root (Jung, 1935a; Von Franz, 1974). This relationship between the endogenous “knowability” of the object world by the brain/mind and mathematical (scientific) knowledge [as in some revealing dreams, like the famous dream by Descartes (Von Franz, 1998)] is the base of the Hintergrudphysik of Pauli (2006).

In general, we disagree with the line of thought deriving from attachment theory, as it interprets psychogenetic development through what we call a situational tilt. In fact, the emerging dispositional scaffolding process that we mentioned earlier, which from perceptual-motor data will metaphorize lofty concepts, is not primarily patterned by early interactions, but actually *uses* meaningful interactions to give shape to primary, endogenous affects into complex emotions. The key to this dispositional process is that perceptual analysis (which, as we have seen in the previous pages, is an active endogenous pattern-forming activity involving the DMS) is primarily activated not by exteroperceptions, but by the SEEKING emotional system originally activated in connection with enteroceptive stimuli. In our dispositional model, affects and to the somatic enteroceptive conditions of the core-Self are represented as images (see also Damasio, 2010). It is actually an affective image schema that carries a fundamentally emotional-cognitive content, which will then confer value to perceptions and which, therefore, precedes outer perception.
